# *Mallotus philippinensis* Muell. Arg fruit glandular hairs extract promotes wound healing on different wound model in rats

**DOI:** 10.1186/s12906-015-0647-y

**Published:** 2015-04-17

**Authors:** Mayank Gangwar, Manish Kumar Gautam, Shivani Ghildiyal, Gopal Nath, Raj Kumar Goel

**Affiliations:** Department of Pharmacology, Faculty of Modern Medicine, Institute of Medical Sciences, Banaras Hindu University, Varanasi, 221005 India; Department of Microbiology, Faculty of Modern Medicine, Institute of Medical Sciences, Banaras Hindu University, Varanasi, 221005 India; Department of Dravyaguna, Government College & Hospital, S.S.V.V., Varanasi, 221002 India

**Keywords:** *Mallotus philippinensis*, Incision, Excision, Dead space, Wound healing

## Abstract

**Background:**

*Mallotus philippinensis* Muell. Arg (MP, Euphorbiaceae) are widely distributed perennial shrub or small tree in tropical and subtropical region in outer Himalayas regions. Since, *Mallotus philippinensis* have been shown to have a number of medicinal values. Hence our present study was to investigate the healing potential of fruit extract in rat wound models.

**Methods:**

The study includes acute toxicity and wound healing potential of 50% ethanol extract of MP fruit glandular hair (MPE). MPE (200 mg/kg) was administered orally, once daily for 10 days (incision and dead space wound) and 22 days (excision wound). MPE was found safe when given to rats upto 10 times of optimal effective dose. Wound breaking strength (WBS) in Incision wound and rate of contraction, period of epithelization and scar area in Excision wound were evaluated. Granulation tissue free radicals (nitric oxide and lipid peroxidation), antioxidants (catalase, superoxide dismutase, and reduced glutathione), acute inflammatory marker (myeloperoxidase), connective tissue markers (hydroxyproline, hexosamine, and hexuronic acid), and deep connective tissue histology were studied in Dead space wound.

**Results:**

MPE significantly increased WBS and enhanced wound contraction, and decreased both epithelization period and scar area compared with control group. MPE was found to decrease free radicals (50.8 to 55.2%, P<0.001) and myeloperoxidase (44.0%, P<0.001) but enhanced antioxidants (41.1 to 54.5%, P<0.05 to P<0.001) and connective tissue markers (39.5 to 67.3%, P<0.05 to P<0.01). Histopathological evaluation revealed more density of collagen formation with minimal inflammatory cells in deeper tissues.

**Conclusion:**

Thus, the study revealed *Mallotus philippinensis* fruit hair extract, safe and effective in wound healing and the healing effects seemed to be due to decrease in free radical generated tissue damage, promoting effects on antioxidant status and faster collagen deposition as evidenced biochemically and histology.

## Background

Wound is a type of injury in which skin is torn, cut or punctured (an open wound), or where blunt force trauma causes a contusion (a closed wound). Wound healing is a multifaceted and protracted phenomenon which repairs the injured tissue completely or partially depending on the severity of wounding. This whole process can be summed up in three overlapping phases inflammatory phase (consisting of haemostasis and inflammation), proliferative phase (consisting of granulation, contraction and epithelialization) and remodelling phase which organized structure with increased tensile strength [1]. Many factors such as inflammatory and immune responses as well as microbial infection have been reported to impair and delay the healing process. Macrophages play very crucial role in wound healing process by releasing various inflammatory mediators viz. myeloperoxidase, cytokines and free radicals which lead to increased reactive oxygen species (ROS) and myeloperoxidase/destructive enzymes-induced tissue damage and decrease in anti-oxidants which help in healing by neutralizing ROS [2,3].

Plants or herbal products play a crucial role for the treatment of wound healing in almost every part of the developing world [4]. In traditional system of medicine, healers provide crude extract to treat skin afflictions including wounds such as sores, bites, burn and lacerations from a range of medicinal plants. It will contribute to healthcare provisional for rural communities [5]. *Mallotus philippinensis* Lam. Muell. Arg (Euphorbiaceae) (MP) are shrubs or small trees which grow on mountain slopes or valleys, limestone hills or river valleys and forests at an altitude of 300–1600 m. *Kamala*, a red colored powder consisting of glandular hairs from its fruit capsule has been used as ant-helminthic, cathartic and many more pharmacological activities in traditional medicine [6,7]. During literature search we noted that MP was used as traditional healers in India. The natives use the powdered fruit to dress the wounds [8,7]. We have also studied that MP fruit extract possessed good anti-inflammatory and analgesic activity which can be important for the healing effect of MP. Therefore, we studied the ethanol extract of MP for its wound healing activity. However, this plant is employed in herpetic ringworm, scabies and other parasitic skin diseases. During our pharmacological investigation, we have also performed antioxidant and free radical scavenging activity of MP extract, which is important for wound healing [9]. Apart from this work, Furomoto *et al.,* [10] reported important phytochemicals like cinnamtannin B-1, protocatechuic acid in ethanol MP bark extract which have the potential effect on the migration of mesenchymal stem cells from the bone marrow or perivascular regions into blood circulation and reported to improve wound healing in mouse. Protocatechuic acid has been shown to promote the migration and proliferation of adipose tissue-derived stromal cells (ADSCs) *in vitro*. These data suggest that the components of MP may provide new therapeutic options for regenerative medicine and could remodel wounded tissues [10]. Therefore, we studied the ethanol extract of MP for its wound healing activity.

Our laboratory has been engaged in evaluating the ulcer protective and wound healing effects of various herbal plants where we have demonstrated important roles of oxidative stress caused by enhanced status of free radicals versus low antioxidant status [11-13]. The present study was therefore, aimed to evaluate the wound healing effect of 50% ethanol extract of MP fruit glandular hair (MPE) by using different *in-vivo* wound healing animal models. The assessments of the wound healing parameters also include study of anti-oxidants, free radicals, acute inflammatory marker, myeloperoxidase, collagen tissue determinants and histological examination for the scientific support.

## Methods

*Experimental animal* Healthy Charles-Foster albino rats (150–200 g) were procured from the Central Animal House (Reg.no.542/02/ab/CPCSEA), Institute of Medical Sciences, Banaras Hindu °University, Varanasi, India. All the healthy pathogen free animals were housed in polypropylene cages in departmental animal house with standard condition (26 ± 2°C temperature and 44–56% relative humidity, light and dark cycles of 10 and 14 h, respectively, for one week before and during the experiments). Standard rodent pellet diet (Pashu Aahar Vihar, Ramnagar, Varanasi) and water ad libitum were provided for animals. All animal experimental manipulations and postoperative care were conducted according to Institute for Laboratory Animal Research, US i.e. guide for the Care and Use of Laboratory Animals [14,15]. The study also was approved by the Institutional Animal Ethical Committee for experimental work (Notification no. Dean/13-14/CAEC/331 dated 20.11.2013). Animals were anaesthetized with pentobarbitone in the dose of 35 mg/kg intraperitoneally in wound models creation while light ether anesthesia by our expert technician in dosing as the excision wound was raw and painful till 12 days post wound. All experiments were performed in clean, but nonsterile conditions. Animals were allowed to breath spontaneously during the surgery. A heating lamp was used to preserve the body temperature at approximately 37°C. All animals were given regular diet and water *ad libitum* on the day of experiments.

*Plant material and preparation of extract*: *Mallotus philippinensis* fruits were collected from Botanical Garden, Department of Dravyaguna, Institute of Medical Sciences, Banaras Hindu University (25.5° N, 82.9° E; elevation, 79 ft/85 m) India. The plant was collected in March to April during fruiting season and was identified, authenticated by Prof. R.K. Asthana Department of Botany, Banaras Hindu University India. A reference voucher number RKA/BOT/Sept.10-12 was assigned to the plant samples and preserved in Department of Botany. The red color glandular hair powder adheres at the surface of shade dried fruits was collected. Approx 500 g of powder was added 1000 ml of 50% ethanol in a round bottom flask and was kept at room temperature for 3 days in shade. The organic fraction was collected and concentrated *in-vacuum* in a rotary evaporator and the residue was dried in desiccators over calcium chloride till further use. The final yield (w/w) of the extract was 11.6%.

*Drug and chemicals* Vitamin E (Merck Ltd., Mumbai, India) and all the other chemicals and reagents were used of analytical grade.

### Acute oral toxicity

Toxicity studies were performed according to Organization for Economic co-operation and Development (OECD) guidelines. Male and nulliparous and non pregnant healthy female rats were used in this study (n=6, 3 each either sex). Animals were observed for 48 h for their neurological changes such as tremors, convulsions, salivation, sleep, feeding behavior and behavioral changes after administration of MPE [16].

#### Treatment protocol

MPE and the standard drug, Vitamin E (VTE) were suspended in 0.5% Carboxy methyl cellulose (CMC) in distilled water. The animals received MPE/VTE, orally once daily with the help of an oro-gastric tube in the volume of 10 ml/kg body weight from day 1, 4 hour after the induction of wounds either for 10 days (Incision and dead space wound study) or 20 days or till the period of complete epithelization (Excision wound study) while, control rats received 0.5% CMC only.

### Wound healing study

#### Linear incision wound model

Animals were divided in five groups with six rats in each group. Group 1 served as control (CMC), groups 2-4 were treated orally with MPE 100, 200 and 400 mg/kg and group 5 was treated orally with VTE (200 mg/kg, positive control). Animals were anaesthetized with pentobarbitone in the dose of 35 mg/kg intraperitoneally. Two paravertebral incisions (6 cm long) were made through the full thickness of the skin on either side of the vertebral column. Wounds were closed with interrupted sutures, 1 cm apart. The sutures were removed on the 7^th^ day. Wound breaking strength (WBS) was measured on the 10th post-wounding day [11]. A preliminary dose response study with MPE indicated 200 mg/kg of MPE as an optimal effective dose and this dose was then selected for further study on excision and dead space wound models.

#### Excision wound model

Excision model was basically used to monitor wound contraction and wound closure time. Animals were divided in three groups (6 animals in each group), group 1, served as control, group 2, received oral MPE (200 mg/kg) while, the 3^rd^ group received oral VTE (200 mg/kg). The back hairs of the animals were depilated by shaving. The circular wound was created on the dorsal interscapular region of each animal by excising the skin with a circular piece of full thickness (~500 mm^2^) was cut off; wounds were left open. The progressive changes in wound area were monitored by drawing traced on 1 mm^2^ graph paper on the day of wounding and subsequently on alternate day till complete healing of wound. Wound contraction was calculated as percentage of the reduction in wounded area and was calculated by a formula given below. Number of days required for falling of the scar without any residual raw wound gives the period of epithelization [12].

$$ \%\ \mathrm{wound}\ \mathrm{contraction} = \mathrm{healed}\ \mathrm{area}/\ \mathrm{total}\ \mathrm{wound}\ \mathrm{area}\times 100 $$

$$ \mathrm{Where}\ \mathrm{healed}\ \mathrm{area}\kern0.5em  = \mathrm{original}\ \mathrm{wound}\ \mathrm{area}-\kern0.5em \mathrm{present}\ \mathrm{wound}\ \mathrm{area} $$

#### Dead space model

This model is basically used to estimate tissue collagen determinants, antioxidants, free radicals and inflammatory marker, myeloperoxidase. Animals were grouped similar to that in excision wound model. Wounds were created by implanting two polypropylene tubes (0.5 × 2.5 cm^2^ each), one on either side in the lumbar region on the dorsal surface of each rat. Animals were sacrificed and granulation tissues formed on the implanted tubes were dissected carefully on 10^th^ post wounding day.

### Wet tissue study

#### Estimation of protein, antioxidants, free radicals and myeloperoxidase

10% homogenate of the wet granulation tissues was prepared in phosphate buffered saline at 4°C and was used for the estimation of protein [17], antioxidants, superoxide dismutase (SOD) [18] and catalase (CAT) [19] and reduced glutathione (GSH) [20]; free radicals, nitric oxide (NO) [21] and lipid peroxidation (LPO) [22]. The assay of SOD is based on the inhibition of the formation of NADH-phenazine methosulphate-nitro blue tetrazolium formazan. CAT measurement was done based on the ability of catalase to oxidize hydrogen peroxide. GSH activity in the homogenate was estimated by the ability to reduce DTNB within 5 min of its addition against blank. LPO levels were estimated in terms of malondialdehyde (MDA) released during lipid peroxidation. Nitrites and nitrates are formed as end products of reactive nitrogen products during NO formation which are measured by using Griess reagent.

For MPO estimation, granulation tissue (5%w/v) was homogenized in 0.5% hexadecyltrimethylammonium bromide (HTAB, Sigma-Aldrich, Co., St. Louis, MO, USA) with 50 mM potassium phosphate buffer, pH 6 [23]. The homogenate was freeze-thawed three times, sonicated for 10 seconds, and then centrifuged at 14000 × g for 45 minutes at 4°C. The resultant supernatant was used for estimation of MPO. A unit of MPO activity is defined as that converting 1 *μ* mol of H_2_O_2_ to water in 1 min at 25°C.

### Dry tissue study

#### Estimation of collagen determinants

Approximately 200 mg wet granulation tissue were collected and dried at 60°C for two days. 40 mg of dry tissue was taken and hydrolyzed using 6 N HCl for 24 h on water bath. The resultant hydrolysate were cooled and neutralized by 10 N NaOH using phenolphthalein indicator which was then finally diluted with water to make final 20 mg/ml concentration of dried granulation tissue. The resultant hydrolysate was used for the estimation of hydroxyproline [24], hexosamine [25] and hexuronic acid [26] and calculated following the standard curve prepared using the proper substrate.

### Histopathology

The deep granulation tissues specimen was collected to examine the histological changes. The samples were fixed in 10% buffered formalin, processed, blocked with paraffin, then sectioned into 5 *μ* m sections, and stained with hematoxylin and eosin (HE) stains. The tissues were examined by light microscope. Fibroblast proliferation mononuclear and/or polymorphonuclear cells, neovascularization and collagen depositions in deeper collagen tissues were analyzed for wound healing in all the groups.

### Statistical analysis of the data

Experimental values are expressed as mean ± SEM (n=6). Wound healing data was statistically performed primary by repeated measure ANOVA to measure changes in each animal before and after treatment while one-way analysis of variance (ANOVA), Dunnett’s test for multiple comparisons between groups. Software’s SPSS Version 16 and Graph Pad Prism, version 4 were used for all statistical analysis.

## Results

### Acute toxicity

For the acute toxicity test, the oral LD_50_ of ethanol extract of MPE were found to be greater than 2000 mg/kg of body weight in both male and female CF rats. At the end of study period (7 days), no death were recorded and appeared active with healthy sign and symptoms. During the day of observation period, the animals were observed no significant sign of toxicity, adverse pharmacological effects or abnormal behavior. This result may indicate that *Mallotus philippinensis* fruit extract has no acute toxicity.

### Wound healing study

#### Incision wound model

WBS was increased dose-dependently by MPE 100, 200 and 400 mg/kg from 323.3 ± 10.5, 368.3 ± 10.1 to 418.4 ± 14.0 (*P*<0.1 to *P*<0.001) compared to control rats which showed WBS as 291.7 ± 14.2 g on 10^th^ post wound day. Standard drug, VTE (200 mg/kg) treated rats showed WBS as 428.3 ± 21.1 g (*P* <0.001). Optimal effective dose of 200 mg/kg of MPE was then selected for further work.

#### Excision wound model

**Figure 1:**
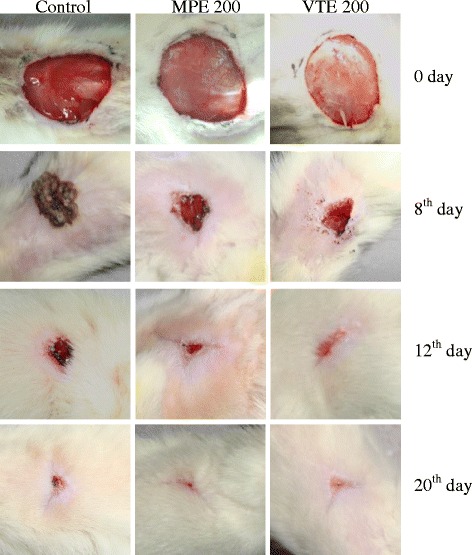
Images represents excision wound healing of control (0.5% CMC), MPE (200 mg/kg) and VTE (200 mg/kg) oral treated rats on day 0, 8, 12 and 20.

Excision model showed a time dependent contraction of rat wound in control rats, while complete epithelization was observed on the 24^th^ day. Contraction rate in control group was 25.7 to 68.3% from day 4 to day 12 while 80.2 to 98% from day 14 to day 20. The average number of days of epithelization and scar area was 10.5 days and 79.5 mm^2^ respectively. Wound contraction percentage of rats treated with both MPE and VTE (200 mg/kg) showed faster and similar contraction of 40.1 and 46.5%, 77.1 and 82.6%, and 99.4 and 99.9% on day 4, 12 and 20 respectively compared with control group (Table 1 and Figure 1). Mean epithelization period and scar area of MPE and VTE were 8.2 and 7.4 days and, 29.2 and 26.3 mm^2^ respectively indicating their comparable effects.

**Table 1:** **Effect of MPE and VTE on wound contraction, epithelization period and scar area in rats: excision wound study**

**Oral treatment (mg/kg, od)**	**Wound area in mm** ^**2**^ **/rat**										
	**Before treatment 0 day**	**After treatment**								***F*** **values** ^*^	***P*** **values** ^*^
		**4** ^**th**^ **day**	**8** ^**th**^ **day**	**12** ^**th**^ **day**	**14** ^**4h**^ **day**	**16** ^**th**^ **day**	**18** ^**th**^ **day**	**20** ^**th**^ **day**	**22** ^**nd**^ **day**		
Control (CMC)	531.2±7.9	432.8±8.9	308.1±15.1	201.3±11.4	125.2±9.1	91.7±4.8	44.7±2.4	18.7±2.4	5.5±0.3	486.4	0.000
	(100.0±0.0)	(74.3±2.4)	(51.2±2.6)	(31.7±2.1)	(19.8±0.9)	(14.4±1.3)	(6.6±0.5)	(2.0±0.5)	(0.7±0.1)		
MPE (200)	521.7±7.7	321.5±4.6^c^	198.5±6.6^c^	114.5±6.7^c^	83.7±4.2^c^	44.2±3.6^c^	20.8±1.4^c^	4.2±0.3^c^	0.0±0.0^c^	1692.0	0.000
	(100.0±0.0)	(59.9±0.9)	(35.0±1.0)	(18.4±1.2)	(13.9±0.7)	(5.7±0.7)	(3.3±0.3)	(0.6±0.1)	(0.0±0.0)		
VTE (200)	543.7±9.6	314.0±8.2^c^	187.8±10.9^c^	103.3±3.9^c^	43.3±5.9^c^	35.3±3.1^c^	6.7±0.6^c^	0.7±0.1^c^	0.0±0.0^c^	926.0	0.000
	(100.0±0.0)	(53.5±1.5)	(25.5±2.1)	(17.4±0.8)	(5.2±1.2)	(4.6±0.6)	(0.9±0.1)	(0.1±0.02)	(0.0±0.0)		

Values are mean ± SEM (n= 6). Values in parenthesis indicate mean ± SEM percent of respective 0 day value using repeated measure ANOVA.

Statistical analysis was done by repeated measure ANOVA^*^ in between the group as indicated by *P* and *F* values in the table while, one way analysis of variance for multiple comparisons was applied between groups (*P* ^c^< 0.001 compared to respective day control group).

### Dead space model

#### Wet granulation tissue parameters

MPE and VTE showed increase in both Wet tissue weight (mg/100 g body weight) 470.8 ± 23.1 and 449.4 ± 13.6 (*P*<0.01) and protein (mg/g wet tissue) 61.1 ± 1.07 and 65.8 ± 2.15 (*P*<0.01) compared to control group wet tissue weight (373.3 ± 15.8 mg) and protein (52.3 ± 2.49 mg).

**Figure 2:**
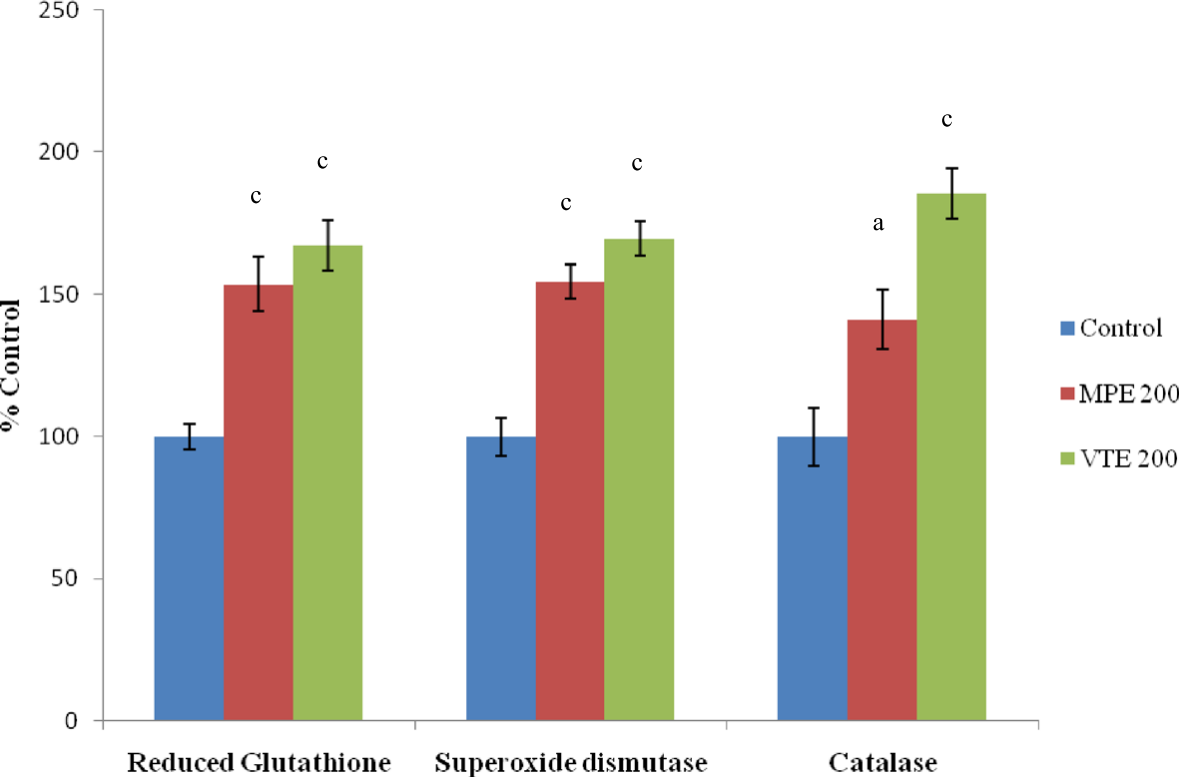
Effect of MPE and VTE on deep granulation tissue antioxidants, reduced glutathione (GSH), Superoxide dismutase (SOD) and Catalase (CAT). Results are mean ± SEM of 6 rats in each group. Control values of GSH, 13.1±0.56 nM/mg protein; SOD, 323.3±21.7 mU/mg protein and CAT, 37.0±3.75 mU/mg protein. P values: ^a^< 0.05 and ^c^< 0.001 compared to respective control group. (Statistical analysis was done by one way analysis of variance followed by Dunnett’s test for multiple comparisons).

**Figure 3:**
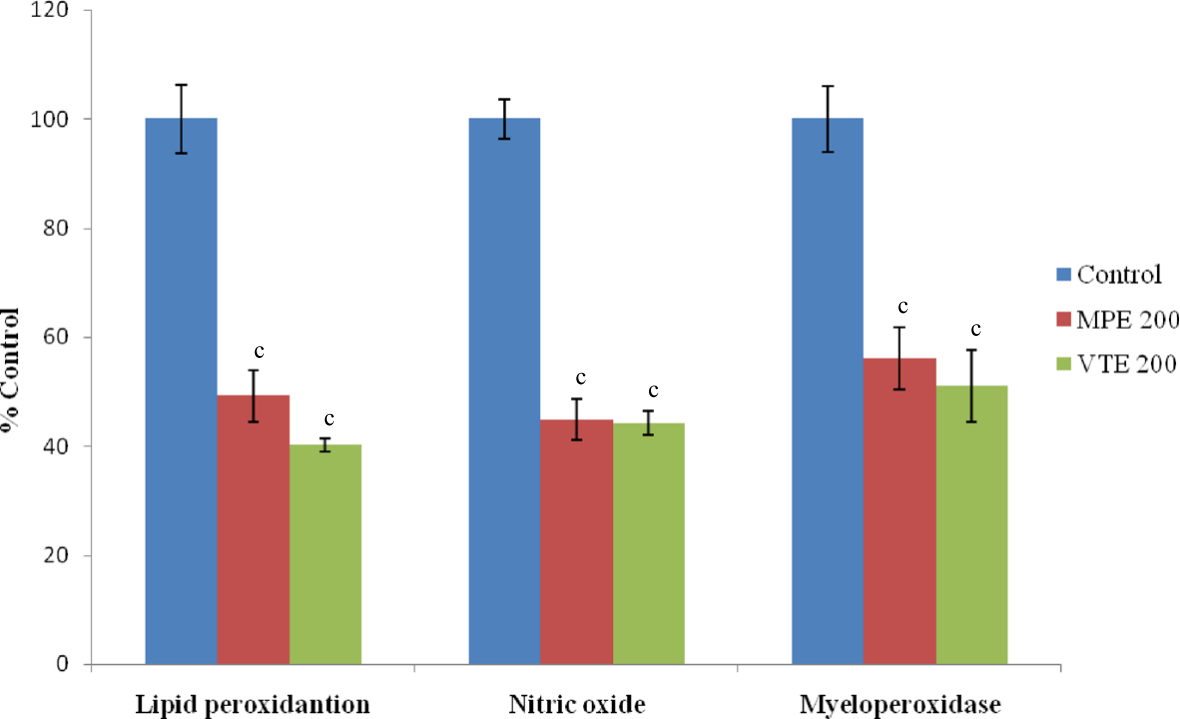
Effect of MPE and VTE on free radicals (Lipid peroxidation, LPO and nitric oxide, NO) and inflammatory marker, myeloperoxidase (MPO). Results are mean ± SEM of 6 rats in each group. Control values of LPO, 5.57 ± 0.35 mU/mg protein and NO., 32.6 ± 1.15 mM/mg protein; and MPO, 30.2 ± 1.79 mU/mg protein. P value ^c^< 0.001 compared to respective control group. (Statistical analysis was done by one way analysis of variance followed by Dunnett’s test for multiple comparisons).

The mean control values for antioxidants, GSH, SOD and CAT were 13.1 ± 0.56 nM/mg protein, 323.3 ± 21.7 and 37.0 ± 3.75 mU/mg protein respectively; free radicals LPO and SOD were 5.57 ± 0.35 and 32.6 ± 1.15 nM/mg protein while, MPO was 30.2 ± 1.79 mU/mg protein. Both MPE and VTE showed increase in the level of antioxidants (*P*<0.001) while, free radicals and acute inflammatory marker, MPO were decreased (*P*<0.001) (Figures 2 and 3).

#### Dry connective tissue parameters

MPE showed a significant increase in the dry weight (15.9%, *P*<0.05) of granulation tissue. Protein (29.0%, *P*<0.05) and levels of collagen determinants; hydroxyproline, hexosamine and hexuronic acid (34.8 to 85.2% increase, *P*<0.05 to P<0.001) were also increased significantly in dry granulation tissue compared with respective control group. The results with MPE were comparable with VTE on the connective tissue parameters (Table 2).

**Table 2:** **Effect of MPE and VTE on deep granulation tissue dry weight, protein, hydroxyproline (HXPR), hexosamine (HXAM) and hexuronic acid (HXUA): dead space wound study**

**Oral treatment (mg/kg, od × 10 days)**	**Dry tissue (mg/100 g bw)**	**Protein (mg/g dry tissue)**	**Connective tissue parameters**		
			**μg/mg protein**		
			**HXPR**	**HXAM**	**HXUA**
Control (1%CMC)	72.2 ± 2.78	178.9 ± 12.8	157.8 ± 8.19	79.9 ± 8.22	18.9 ± 2.29
	(100.0 ± 3.85)	(100.0 ± 7.15)	(100.0 ± 5.19)	(100.0 ± 10.3)	(100.0 ± 12.1)
MPE 200	83.7 ± 2.51^a^	230.8 ± 14.4^a^	220.1 ± 12.5^b^	133.7 ± 13.1^b^	27.2 ± 1.47^a^
	(115.9 ± 3.48)	(129.0 ± 8.05)	(139.5 ± 7.92)	(167.3 ± 16.4)	(143.9 ± 7.78)
VTE 200	86.6 ± 2.76^b^	232.7 ± 11.4^a^	212.7 ± 9.40^b^	129.1 ± 9.37^b^	35.0 ± 2.00^c^
	(119.9 ± 3.82)	(130.1 ± 6.37)	(134.8 ± 5.96)	(161.6 ± 11.7)	(185.2 ± 10.6)

Results are mean ± SEM (n=6). Values in parenthesis indicate percent of respective control value.

*P* values: ^a^< 0.05, ^b^< 0.01 and ^c^< 0.001 compared to respective control group (Statistical analysis was done by one way analysis of variance followed by Dunnett’s test for multiple comparisons).

**Figure 4:**
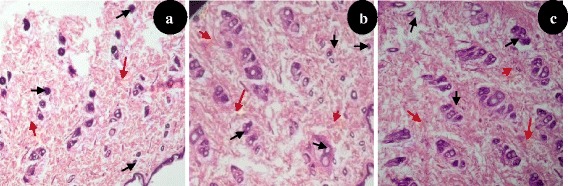
Histopathological examination of dead space granulation tissue at day 10 stained with H&E (100 x). **(a)** granulation tissue of control rat showed mononuclear inflammatory cells (black arrow), scattered abundance of eosinophilic fibroblasts (red arrow). **(b)** MPE treated showing large number of collagen tissue (fibrosis) and neovascularisation with minimal inflammatory cells. **(c)** VTE treated showing near to normal features, collagen tissue (fibrosis) and neovascularisation.

Histology of deep granulation tissue of control rat showed scattered eosinophilic collagen tissue and some mononuclear inflammatory cells while, MPE and VTE treated rats showed large number of collagen tissue, neovascularisation with minimal mononuclear inflammatory cells (Figure 4).

## Discussion

Healing of wound occurs mainly in three major processes i.e. epithelization, contraction and connective tissue deposition. New collagens and its subsequent maturation decide and regulate the rate and extent of healing [11]. In the early process of wound healing, inflammatory cells promote migration and proliferation of endothelial cells, which synthesize extracellular matrices including collagen, and of keratinocytes resulting to neo-vascularization and re-epithelialization of the wounded tissue [27]. Collagen and collagen derived fragments regulates different cellular functions such as cell shape and its differentiation, migration and synthesis of a number of proteins in extracellular matrix [28]. Strength of wound depends on remodeling of collagen and the formation of stable intra- and inter-molecular cross links. Large fibrils and complex fibrous superstructures are formed from collagen which is mainly responsible for tensile strength of the tissue [29,30]. In incision wound model, MPE treated group showed an increase in tensile strength which may be attributed due to the increase in collagen concentration and stabilization of the fibers [11]. Wound contraction involves contraction, narrowing or closing of the wound. Process of healing is regulated by synthesis, deposition and maturation of collagen [31]. MPE treated group showed faster contraction of wound might be due to interleukin-8 stimulation, an inflammatory *α* -chemokine which alters the function and recruitment of various inflammatory cells, fibroblasts and keratinocytes [32].

Collagens breakdown results in liberation of free hydroxyproline which can be a good index for measuring collagen turnover in the granulation tissue. The result showed that MPE treated rats showed an increase in hydroxyproline level causing rapid healing with concurrent increase in breaking strength. Along with hydroxyproline, hexosamine is the matrix molecule which acts as a ground substance for new extracellular matrix in wound area. There are various reports which suggested increased level of hexosamine resulted into stabilization of collagen molecules by enhancing electrostatic and ionic interaction [33]. These molecules have the ability to bind and alter protein-protein interaction that plays a key role in cellular responsiveness in development, homeostasis, and disease [34]. MPE was found to increase the level of hexosamine reflecting its wound healing effects.

Oxidative stress is responsible for the production of reactive oxygen species (ROS), which results in delayed healing process and cytotoxicity [35]. During chronic wound, along with oxidative stress, neutrophil derived peroxidases including MPO and cytokines contribute in tissue damage [36]. The main strategy for healing the chronic wound is thus, to eliminate the over produced ROS [37]. Therefore, enhanced levels of antioxidants would lead to ROS elimination and inflammation (due to decreased oxidative stress) and better chances of wound healing. Our experiment on wound healing in rat treated with MPE showed increased antioxidants but reduced myeloperoxidase and free radical levels which promoted the healing process by preventing inflammation and oxidative stress.

*Mallotus philippinensis* (MP) is widely used as a traditional medicine in several parts of countries including India. MP contains the active phenolic compounds, di and triterpenoids, steroids, flavonoids, coumarinolignoids, phloroglucinol derivatives, benzopyrans etc. which are responsible for their various biological activities like anti-inflammatory, wound healing, antioxidant etc. Rottlerin, also called mallotoxin is an active compound present in MP was shown to have antioxidant, antiradical, anticestodal [38,39]. Bark of Mallotus has been reported for total antioxidant activity and antiradical effect against DPPH, and phenolic fraction showing strongest antiradical activity against DPPH and reducing power [40]. Different Bark extract of *Mallotus philippinensis* has been tested *in-vitro* for wound healing activity by examining the proliferation, migration of mesenchymal stem cell by secreting various cytokines to wounded site from bone marrow to systemic circulation and finally remodel wounded tissues [10]. The beneficial effect of MPE on wound healing could be attributed to the presence of many active principles promoting wound healing through their antioxidant, anti-inflammatory and effects on inflammatory cytokines and peroxidase.

Wound while exposure to external environment becomes prone to attack by microbes which would alter the natural healing process. MP has been shown to have wide range of antimicrobial activity against human pathogens which could reduce the skin infection and enhance wound healing process [41].

## Conclusion

The result of the study supports the traditional use of *Mallotus philippinensis* fruit for wound healing potential. *Mallotus philippinensis* fruit glandular hair extract demonstrated significant wound healing activity through enhancing collagen synthesis and antioxidant effects neutralizing the tissue damaging effects of free radicals and inflammatory cytokines and damaging peroxidase enzymes as evidenced from the histological and biochemical studies of deep granulation tissue. Thus, the present investigation scientifically validates the use of *Mallotus philippinensis* in wound healing.
